# On Typhus or Ship Fever, as Witnessed at Grosse Isle

**Published:** 1848-04

**Authors:** 


*On Typhus or Ship Fever, as witnessed at Grosse Isle.* By Geo. M. Douglas, M. D., Med. Sup., Quarantine Establishment, Grosse Isle.—Typhus and Typhoid Fever, popularly known at different periods, from its prevalence in particular localities, as *Jail*, *Camp*, *Hospital*, *Ship*, *Road*, or *Irish* Fever, is unquestionably one of the oldest diseases of which mention is made by historians. From the earliest ages, it has been remarked, that the accumulation of ill-fed people, in situations where ventilation and cleanliness are neglected, generates fever of a malignant type, which propagates itself in a manner more certain than any other disease. Thucydides ascribes the great plague of Athens to the introduction by Pericles of multitudes of rustics into the city, and who were crowded together in huts within the walls ; and Livy imputed the first great plagues of Rome to the number of inhabitants pent up within its narrow limits. Medical writers are agreed in attributing to these causes the great plagues that devastated the city of London at different periods. Defoe, in his History of the Plague, very wisely counsels the authorities, "that they would consider of separating the people into smaller bodies, and remove them in time farther from one another, and not let such a contagion as this (plague), which is indeed chiefly dangerous to collected bodies of people, find a million of people in a body together again." The great fire of London did this for them the following year. The progress of civilization, the improvement in the moral and physical condition of the masses, and the great and increasing attention paid to cleanlines, ventilation, and drainage, together with a more regular and certain supply of food, have tended to remove many of the causes of fever in Europe. In Ireland we still find it indigenous ; and though I am not prepared to go the length of Dr. Lombard, who asserts that the freize coat of the Irish labourer is its depository and vehicle, yet we have in this country abundant proofs that Typhus Fever is every year imported by the Irish emigrants, and by no other. Mr. Farr, the Register-General, has also shown, that in the three great "avenues by which the Irish labourer enters England, viz., Bristol, Liverpool and Glasgow, their crowding to excess in lodging-houses, their loathsome diet and filth, are productive of fever in these cities." And remarks at the same time, "that in thus directing attention to a weighty sanatory fact, it is far from our intention to convey any reflection upon the Irish people, as it can be shown that a few years back the English were as bad."—*MacCulloch's Statistics.*

A reference to the Tables annexed to this, will exhibit a yearly importation of fever into Canada by emigrants. The greater or less prevalence of the disease in Ireland, has been observed to depend on the crop of the common food of the people, the potato. The almost total failure of this esculent for the last two years, has produced a scarcity amounting to famine in some parts, and has thus augmented, to a degree hitherto unknown, the usual concomitant of famine, *fever*. Hence we witnessed last year the melancholy sight of every passenger vessel, with Irish emigrants, arriving freighted with this disease. The same was seen at all the ports of this continent where emigrants landed. The greater mortality in vessels coming to the British Provinces, may be attributed principally to three causes : 1st, the greater length of the voyage ; 2d, the character of the passengers ; and 3d, to being more crowded.

1st, The average length of a passage to Quebec may be estimated at one-third more than to New York, in consequence of the tediousness of the Gulf and River navigation and the inferior class of vessels employed in the timber trade.

2d, The higher rate of passage, and the restriction imposed by the system of bonding in New York, have the effect of driving all the aged, sickly, poor, and destitute to seek the route of the British Provinces. And

3d, The law limits, in the United States, the number to one adult to every fourteen superficial feet, and counts all souls on board. The English Passenger Act limits the number to one adult to every ten feet, and permits infants under twelve months to pass free. These causes will explain the greater mortality and *morbility* of passengers arriving in the British Provinces.

The chief circumstances which tend to render fevers communicable from one person to another are found to be,

1st, Humidity of the atmosphere.

2d, Deficient supply of food.

3d, Filth and imperfect ventilation.

It would be difficult to find any place where these three circumstances are combined in such a degree as in the hold of an Irish passenger vessel. Firstly, in addition to the usual dampness of a ship, you have in an emigrant vessel the humidity caused by the daily distribution of fresh water in small quantities to each individual passenger, and which, being kept by them in tins and pots in and under their sleeping berths, gets capsized very often by the roll of the ship, and thus adds to the general dampness ; and in ill-regulated vessels, the passengers are permitted to wash their clothes in the 'tween decks. The air of the hold is thus always sur-charged with moisture, while its temperature is kept up by the heat given off by an accumulation of living bodies.

2ly, The supply of food is in many cases limited to a pound of bread-stuffs, or oatmeal, to each adult per diem, and this often in a mouldy damaged state. Thousands of the emigrants who arrived in Canada last season, had no other sustenance on the voyage.

3dly, The peculiar nature of a ship's hold is such, that ventilation, while the ship is under weigh, is all but impossible, in the only way in which perfect ventilation is obtained, viz: by passing a current of pure air *through* the hold. Wind-sails effect this in a very insufficient manner ; and when, in rough weather, it becomes necessary to fasten down the hatches, the little supply of air which enters by them is shut out. Of the passengers a great portion are women and children, who are unable, in many instances when the weather is stormy, to avail themselves of the miserable "*cabinets d'aisances*," (as when they do exist they are generally in the bows of the ship,) and are consequently obliged to pass their evacuations in the hold. You have here combined, all that could by any possibility generate a foul atmosphere ; and when to this you have febrile miasma, the only wonder is that any escape the disease, as there is no running away from it. On visiting a passenger vessel, such as thus described, in a morning before the emigrants have come on deck, I have seen a stream of foul air issuing from the hatches as dense and palpable as seen on a foggy day from a dung heap or range of hot-beds ; and rarely did I find it necessary to enquire if fever prevailed on board ; that peculiar and characteristic odor which belongs Typhus Fever patients was perceptible to the sense on stepping on deck. To the foregoing cases ought to be added, the moral and depressing influence of fear of shipwreck, and grief at leaving their native land—both powerfully predisposing causes of fever.

The character of the disease as witnessed at Qarantine Hospitals, did not differ essentially from that so often and so well described in this country, as well as in Europe. The three great systems were more or less affected in all cases. In some instances the organs constituting the nervous system were more prominently affected ; such cases were more frequently seen in the better fed seamen, or English emigrant, who were occasionally found mixed up, in Liverpool vessels, with the Irish. In these cases the disease was ushered in with intense headache, great pain in the back and limbs, bloodshot eye, and early furious delirium. In some rare instances the sensorial faculties were overwhelmed at once as completely as in apoplexy. A stout healthy young man of 18, was struck down with such an attack on board the barque "Gilmour," in which vessel he was an apprentice : he expired in twelve hours. A similar case was witnessed in the ship "Mail," from Liverpool ; a stout seaman was attacked, and death supervened with equal rapidity. Both these vessels were unusually sickly. These cases are cited to prove the power of concentrated miasm acting on the nervous system.

The organs of secretion and excretion were more frequently affected than any other ; and such cases were invariably found most troublesome to treat, and more frequently had a fatal termination. Frequent observation convinced us of the correctness of Dr. Cheyne's remark, that dysentery was sometimes converted into fever, while *vice versa*, fever was converted into dysentery. When the symptoms of fever were exchanged for those of dysentery, it was probably by the irritation of the mucous coat of the small intestines and stomach extending to the large. Sydenham expressed the opinion, that dysentery is a *febris introversa*, or turned in upon the intestines.

Cases in which the derangement of the circulating system predominated, were not of such frequent occurrence as compared with others.

*Petechiæ* and maculæ were found in many cases, but were not so constant and universal as to justify our classing Typhus in the list of exanthems. Epistaxis was a troublesome accompaniment, and such cases where they did not terminate fatally, had a long and tedious convalescence. Dr. Benson, one of the medical attendants at the Quarantine Hospitals last season, a gentleman advanced in years, and for a long period connected with a large fever hospital in Ireland, fell a victim to an attack in which great hæmorrage from the nose and fauces was a prominent symptom. On its advent he resigned himself to death, saying he had never seen such cases do well in elderly people. He died on the fourteenth day.

Meteorism, or tympanic swelling of the belly, was another unfavorable symptom, occasionally met with towards the close of fever. A medical assistant in the Hospitals in 1836, who died from Typhus, had this distressing affection supervene on the seventeenth day. In this case, it was ascertained by a subsequent post mortem examination, to have been caused by ulceration of the ileo-cœcal valve. Forty-eight hours previous to his dissolution, there was as complete a reversion of the peristaltic motion, as in the most perfect case of strangulated hernia, as evinced by constant vomiting of fecal matter. Severe and protracted cases, especially those which ran into dysentery, were often complicated with excoriation and sloughing sores of the back, sacrum, and hips; the irritation and exhaustion produced by these, frequently turned the scale against the unhappy sufferer. Various topical applications were made to these sores, and means adopted to take off pressure. Among the former of these was a weak solution of Mr. Ledoyen's disinfecting fluid (solution of nitrate of lead). Marked benefit was obtained by its use in many cases, and in all it corrected the effluvium which so constantly attends large sloughs. In two of the cases where it was so used, it brought on lead colic.

Inflammation and swelling of the parotid gland was an occasional event after the fifteenth day; it was always looked upon as an unfavorable symptom, terminating often with gangrene and death with us.

Among the irregular and anomalous cases which occurred, were two or three, in which there existed an enormous craving for food, chiefly for meat, and any kind would be swallowed with avidity. This took place in each instance during the height of the fever, and at a time when the tongue was dry, cracked and glazed, the face flushed, and the pulse over 100. A similar phenomenon was observed during the progress of the fever with which Dr. Painchand, Sen. Physician of the Marine and Emigrant Hospital of this city was attacked. In this case, during the ingravescence of the disease, and at various periods afterwards, he was seized with an inordinate desire for food, which he eat with avidity, swallowing large pieces of beef steak imperfectly masticated. His convalescence was tedious, and accompanied by diarrhœa. I find that Dr. Satterley relates an analogous case, in the Medical Transactions, vol. v. Art. xxii., where the desire for food re-appeared on the fifth day, with a craving which it was impossible to satisfy. When the food was not allowed, various indigestible substances were devoured in its stead. In this case the disease extended, with numerous variations, to upwards of thirty days, when the fever unequivocally subsided, and the patient gradually recovered. A greater number of deaths took place from relapse after fever and from dysentery, than from fever itself; in both instances induced by errors of diet, which the greatest watchfulness could not prevent; or from sudden atmospheric changes, to which the convalescents were exposed in hospitals built of boards, and constructed with a view to free ventilation. The treatment was necessarily modified in different cases, according to the predominance of diseased action, in the different organs, whether in the *brain*, *chest*, or *abdomen*.

General bleeding was rarely employed, as few cases were seen in the very outset, when this remedy, if used at all, is alone justifiable. I fully concur with Dr. Smith in the opinion, that "it is in vain to hope to terminate fever by a stroke of art; that when the disease is once set up, it advances with a step as steady as time, and, like time, it never retraces a step." Many of the medical gentlemen in charge of the hospitals at Grosse Isle last season, had great faith in emetics in aresting the disease, but all sooner later gave up the their use, from a conviction of their inefficacy. Cleanliness, quietness, cool drinks, gentle aperients of calomel and rhubarb, or senna and salts, so as to produce two or three stools in the twenty-four hours, with three half pints of gruel or arrowroot per diem for diet, were the chief means resorted to during the progress of the fever, If head symptoms showed themselves, the *douche* was used, and a single fold of linen cloth wet with cold water was kept applied to the shaved scalp. If there still existed great restlessness and insomnia notwithstanding these applications, recourse was had to hyosciamus, as long experience has taught me, that delirium, coma, and death, often ensue, where attention to the important point of obtaining sleep is neglected. Stimulants were rarely employed in the early stages of the disease; towards the close and when the struggle came, brandy and wine were freely used, and when these failed to rouse the sinking powers, great benefit was often derived from the administration of large doses of gum-camphor; dose of 20 to 30 grains three times in the twenty-four hours were given, in substance reduced to a powder by means of a drop or two of spirits of wine. I have witnessed the most astonishing effects from the use of this drug, in cases where there was almost total insensibility, a thread-like pulse and complete loss of muscular power, as evinced by the sliding down in the bed. In such cases, re-action has been brought on, and the flagging powers recalled by it, even when wine and brandy by the half pint had failed to stimulate. Tartar emetic was used with benefit where the disease showed itself in the chest.

In the abdominal affection, where there was much purging, starch enemata with laudanum was administered, and a rag wet with turpentine was applied externally. This form of disease was always the most troublesome and unmanageable, frequently baffling all the curative means employed. *Alum*, the *mineral acids* combined with opium, chalk with and without opium, and the whole catalogue of astringents, were tried by the young medical men fresh from the schools, and having great faith in drugs. I did not find one who was not disgusted sooner or later with his pet remedy.

The disease is both contagious and infectious. In proof of which I need only refer to the Table (2) appended to this, to show how unerringly it was contracted by those whose duties brought them in contact with the sick in hospitals and ships. On the other hand solitary cases placed by themselves, where strict attention was paid to cleanliness and ventilation, frequent change of the clothes of the person and bed, and the immediate removal of all *ejecta*, appeared to be incapable of generating febrile miasm in sufficient quantity to communicate the disease. I only knew of one person so contracting fever last year among people in easy circumstances. Those who have once had an attack (with few exceptions) appear to possess a certain immunity for years after. I suffered in my own person from a severe attack, with petechial eruptions, delirium, and other bad symptoms, in 1836, and, though exposed every season since, have hitherto escaped a second attack. When exposed to concentrated miasm in the holds of sickly ships, or when the hospitals are much crowded, and the weather calm and sultry, I suffer from derangement of the bowels, lassitude, and nausea.

With respect to the morbid appearance after death, I regret that the want of room, time, and other difficulties, prevented advantage being taken of the extensive field of observation presented last year at the Quarantine Hospitals. Previous to last season I made a point of examining all bodies; on reference to notes then taken, I find the following morbid appearance more frequently met with: In the head the dura mater was rarely found diseased, though frequently adherent to the calvarium; the arachnoid and pia mater were often found more vascular than in a normal state, the substance of the brain itself, on being sliced with the scalpel, presented more bloody spots than usual; and there was in all cases effusion more or less into the ventricles, in some instances to the extent of two or three ounces. In the chest, the lining membrane of the bronchi was invariably found highly vascular, and thickened; and frothy mucus mixed with pus was found in the smaller tubes. The pleuræ were often found inflamed, and adherent in all cases at one or more points. The lungs were rarely found in a normal state, being either engorged with blood or serum, or presenting indications of inflammation, such as hepatization, &c. The heart and pericardium were seldom found to offer any morbid appearance. In the abdomen most of the organs, except the liver, were generally found deviating from a healthy condition.

The lining membrane of the intestines was found in various states of disease, from simple increased vascularity to positive ulceration. This latter morbid appearance was more often found in the large intestines, about the ileo-cœcal valve; increased vascularity and inflammation were rarely met with in the membrane of the stomach.

Where the large intestines were found in an ulcerated state, enlargement and induration of the mesenteric glands were remarked, especially of those attached to the diseased intestine.

[We omit Table 1, shewing the number and per centage of disease, and of deaths of Emigrants at the Quarantine Hospital, Grosse Isle, from 1833 to 1847, both years inclusive.]

TABLE 2.

Showing the number of Clergy, Medical Men, Hospital Attendants and others who contracted Fever and died during the last season in attendance upon the Sick Emigrants at Grosse Isle.

| | Number who attended Hospital. | Number who contracted Fever. | Number who died. |
|---|---|---|---|
| Roman Catholic Priests | 42 | 19 | 4 |
| Clergymen of the Church of England | 17 | 7 | 1 |
| Medical Men | 26 | 22 | 4 |
| Hospital Stewards | 29 | 21 | 3 |
| Nurses, Orderlies and Cooks | 186 | *76 | 22 |
| Policemen | 10 | 8 | 3 |
| Carters employed to remove the sick, dying and dead | 6 | 5 | 2 |
| Clerks, Bakers, and Servants of Mr. Ray, suttler | | 15 | 3 |
| Do. of Mr. Bradford. | | 4 | 1 |
| Deputy Emigrant Agent | 1 | 1 | |
| Clerk to ditto | 1 | 1 | |
| Custom House Officers employed to examine baggage | 2 | 1 | |
| Servants of Roman Catholic Clergymen | 8 | 4 | 1 |

TABLE 2.

Showing the average daily number of sick during each month of the season:—

| | |
|---|---|
| May 15th to 31st | 451 |
| June 1st to 30 | 1508 ½ |
| July 1st to 31st | 1454 ¾ |
| August 1st to 31st | 2021 1-3 |
| September 1st to 30th | 1330 1-30 |
| October 1st to 21 | 346 9-31 |

Average daily number sick during the season, 1307 8-159.

[*British American Journal.*

---

*Warm Bath in Dropsy after Scarlet Feverr.*—Dr. Golding Bird emarks, that as a prophylactic remedy the warm bath is invaluable n the treatment of dropsy after scarlet fever. He scarcely recol-

---

* Many of the Hospital Orderlies, Nurses, and Cooks were Emigrants, who were employed after their convalescence from fever, otherwise the proportion of sick would have been greater ; as nearly all those who came down from Montreal and Quebec to be engaged, contrrcted fever, either at Grosse Isle or soon after leaving it.

